# Highly Polarized Single Photons from Strain-Induced Quasi-1D Localized Excitons in WSe_2_

**DOI:** 10.1021/acs.nanolett.1c01927

**Published:** 2021-08-23

**Authors:** Qixing Wang, Julian Maisch, Fangdong Tang, Dong Zhao, Sheng Yang, Raphael Joos, Simone Luca Portalupi, Peter Michler, Jurgen H. Smet

**Affiliations:** † Max Planck Institute for Solid State Research, Stuttgart D-70569, Germany; ‡ Institut für Halbleiteroptik und Funktionelle Grenzflächen, Center for Integrated Quantum Science and Technology (IQST) and SCoPE, University of Stuttgart, Stuttgart D-70569, Germany

**Keywords:** quasi-1D localized excitons, linear polarization, valley hybridization, circular polarization

## Abstract

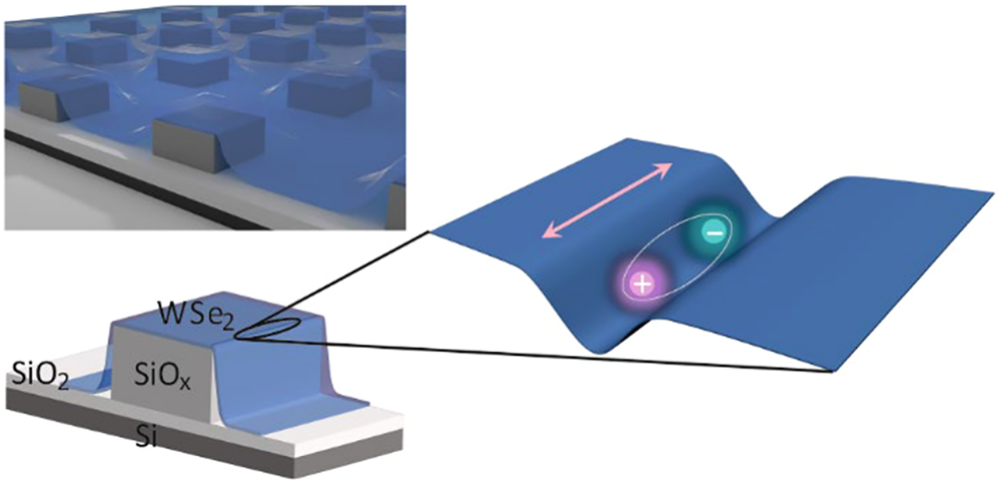

Single photon emission from localized excitons in two-dimensional (2D) materials has been extensively investigated because of its relevance for quantum information applications. Prerequisites are the availability of photons with high purity polarization and controllable polarization orientation that can be integrated with optical cavities. Here, deformation strain along edges of prepatterned square-shaped substrate protrusions is exploited to induce quasi-one-dimensional (1D) localized excitons in WSe_2_ monolayers as an elegant way to get photons that fulfill these requirements. At zero magnetic field, the emission is linearly polarized with 95% purity because exciton states are valley hybridized with equal shares of both valleys and predominant emission from excitons with a dipole moment along the elongated direction. In a strong field, one valley is favored and the linear polarization is converted to high-purity circular polarization. This deterministic control over polarization purity and orientation is a valuable asset in the context of integrated quantum photonics.

## Introduction

The quantum emission of localized excitons serves as an essential building block of quantum optics^1,2^ and quantum information technology.^3,4^ Excitons localized by atomic defects or by a topography-induced dot-shaped strain potential in WSe_2_ have been reported to typically exhibit single photon emission characteristics.^5−9^ The emission of two-dimensional (2D) WSe_2_ can be easily tuned by electric field,^5^ magnetic field,^6,8^ and mechanical strain^10−18^ because it is both atomically thin and flexible.^19,20^ Moreover, it is straightforward to integrate WSe_2_ quantum emitters with plasmonic structures^21^ and photonic waveguides.^22,23^ Therefore, quantum emitters based on WSe_2_ represent a potentially powerful platform for the exploration of quantum photonics concepts. However, emitters must also offer indistinguishability, brightness, and either well-defined spatial modes or polarization modes to be of use in quantum information technology.^24,25^ At present semiconductor quantum dot technology that has matured over decades is well ahead to fulfill such key requirements.^1,24,26,27^ While progress in achieving these with localized excitons from WSe_2_ and more generally 2D materials is rapid, a significant amount of work remains to be done.

In this report, we focus on the engineering of the polarization purity and polarization orientation of WSe_2_ based single photon emitters. We intentionally introduce an extreme asymmetry in the confinement potential to obtain quasi one-dimensional excitons. The quasi one-dimensional nature effectively suppresses the emission of the fine structure split state at higher energy. As a result, single photon emission comes with high purity linear polarization and the orientation is determined by the geometry of the intentionally imposed strain potential. The linear polarization is attributed to valley hybridization, and this is corroborated by a magnetic field-dependent study.

## Results

**Figure 1:**
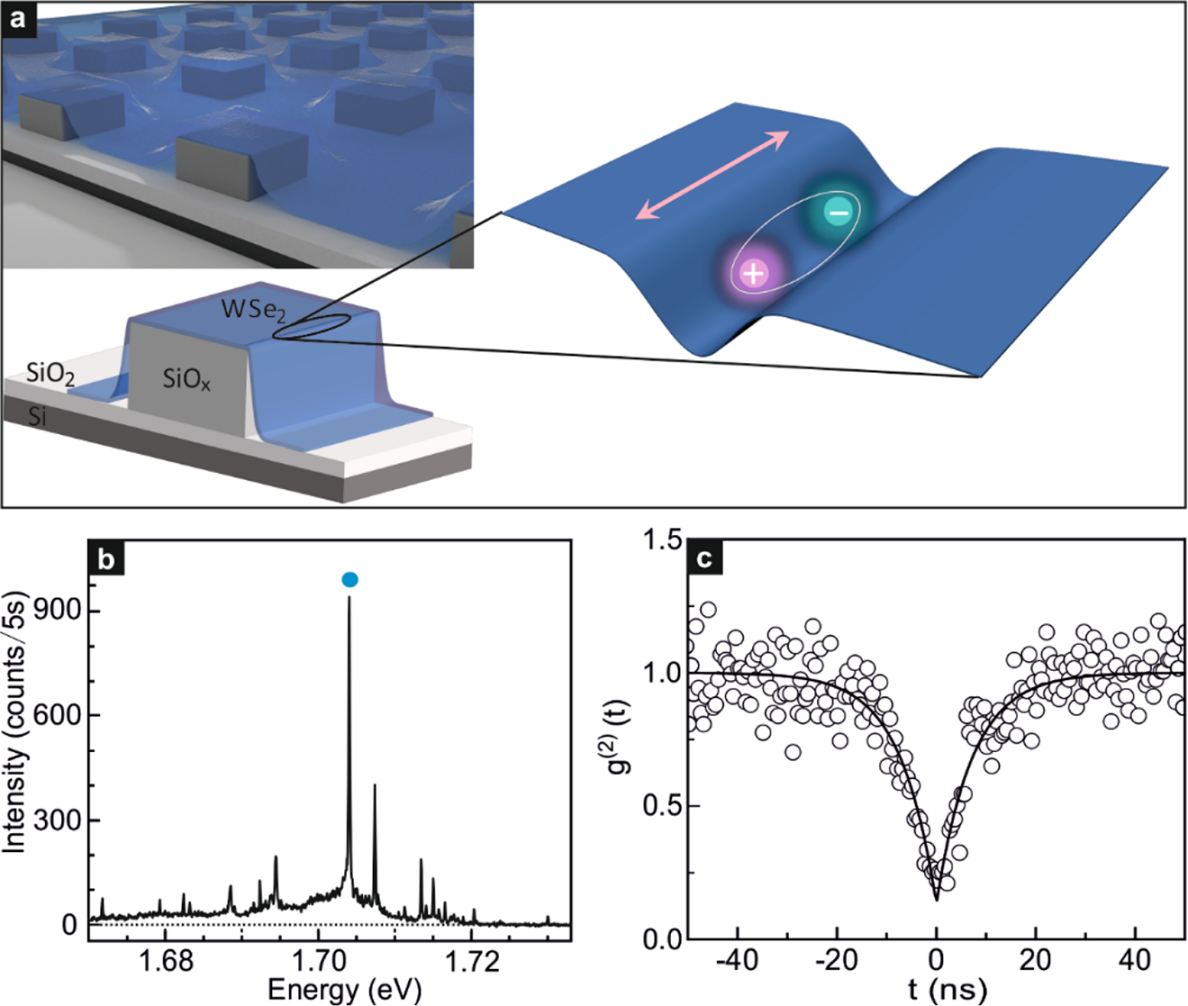
Sample geometry for generating quasi-1D localized excitons. (a) Schematic cross section of a WSe_2_ monolayer placed on top of an array of SiO_*x*_ pillars (left) and schematic illustration of a quasi-1D localized exciton trapped by the strain-induced potential at the edge of the SiO_*x*_ pillar upon laser illumination (right). The arrow marks the orientation of the oscillating exciton dipole. It is parallel to the one-dimensional strain-induced potential along the pillar edge. (b) Micro-photoluminescence (μ-PL) spectrum obtained when illuminating the WSe_2_ monolayer at the edge of SiO_*x*_ pillar with 633 nm laser. The blue dot highlights the emission peak at 1.704 eV investigated in this Letter. It stems from localized bright excitons. The experimental data were recorded at approximately 2 K. (c) Second-order photon-correlation measurement of another localized exciton emission peak, measured near 4 K when pumping with a CW laser with a wavelength at 658 nm. The black solid line is a fit to the data. At zero time delay, *g*^(2)^(0) = 0.13 ± 0.04.

To generate a quasi one-dimensional confinement potential that traps excitons, the WSe_2_ monolayers are clad over an array of square shaped silicon-oxide pillars, each with a footprint of 1 μm × 1 μm. The sample configuration is illustrated in Figure 1a and in Section 1 of the Supporting Information (SI). The strain experienced by the WSe_2_ is estimated to be about 0.5% based on an analysis of the Raman spectra (Section 2, SI). The strain-induced potential varies rapidly perpendicular to the edge of the pillar as schematically shown in Figure 1a and only weakly when moving along the edge until a corner is approached. As a result of this strong asymmetry, excitons whose dipole moment oscillates along the edge (see Figure 1a) are more favorable and have lower energy.

Figure 1b presents an exemplary micro-photoluminescence (μ-PL) spectrum at a temperature of 2 K acquired at the edge of a SiO_*x*_ pillar once covered with a WSe_2_ monolayer. The sharp emission lines originate from the excitons trapped in the strain-induced potential. The peak with the strongest intensity marked by the blue dot is then investigated in more detail. It is centered at about 1.704 eV and has a full width at half-maximum (FWHM) of 161 ± 14 μeV (Section 3, SI). Such narrow line width is consistent with previous reports of WSe_2_ single photon emitters.^5−9^ Second-order photon-correlation measurements were performed on several distinct emitters at 4 K to confirm single photon emission. One example is plotted in Figure 1c and additional measurements can be found in Section 4 of the SI. Data were fitted with the second-order photon-correlation function *g*^(2)^(*t*) = 1– (1 – *a*)*e*^–|*t*|/τ^, where *t* is the time delay between the coincidence counts, τ consists of pumping and radiative recombination rates, and *a* represents the value of *g*^(2)^(*t* = 0). At zero time delay, the correlation function displays pronounced photon antibunching behavior with *g*^(2)^(0) = 0.13 ± 0.04 < 0.5.^5−9^ This unequivocally proves that the WSe_2_ localized excitons act as single photon emitters. The excited-state lifetime, assuming low pumping power as employed in this measurement, would result in an emitter lifetime close to 7.25 ± 0.46 ns. This lifetime falls in the range of previously reported lifetime values (0.5–8.8 ns) of WSe_2_ quantum emitters.^5,7−9,28^

**Figure 2:**
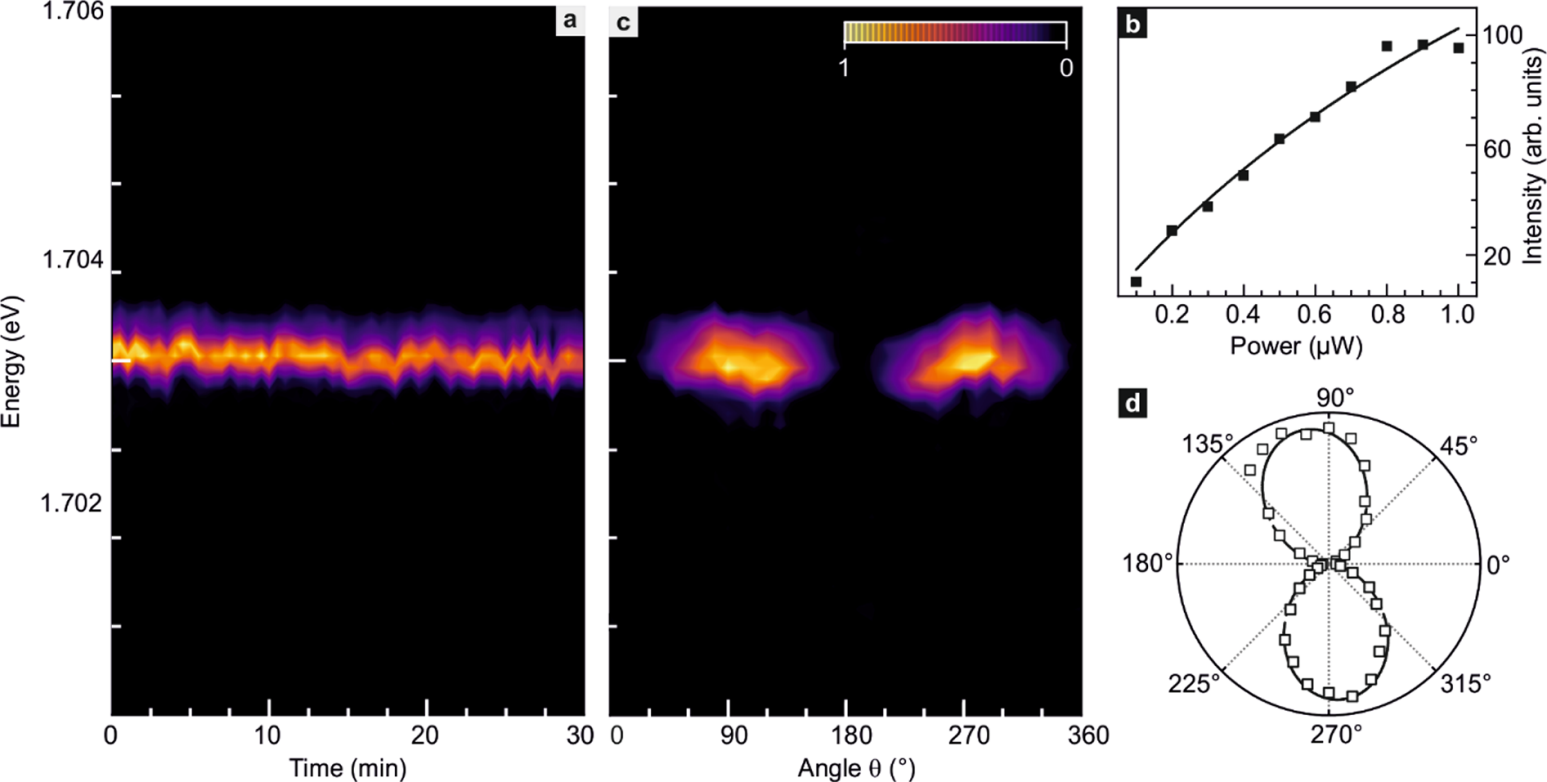
Properties of the emission peak around 1.704 eV. (a) Time dependence of the μ-PL emission feature near 1.704 eV. (b) Integrated μ-PL intensity as a function of the incident laser power. Dots are experimental data points. The line is a fit with the formula . (c) Linear polarization dependence of the μ-PL emission as a function of the detection angle. The plotted quantity is the intensity normalized by the maximum recorded value. (d) Integrated μ-PL intensity near 1.704 eV plotted as a function of the detection angle in a polar diagram. The solid line is a fit to data with the formula . The degree of linear polarization reaches 95%.

The emission spectra of localized excitons in 2D materials may exhibit photobleaching and spectral jitter.^7,8^Figure 2a plots the emission energy and intensity over time for the spectral feature at ∼1.704 eV in order to assess whether this also applies to the emission spectrum of this strain-induced exciton. Photobleaching is absent during the covered 30 min time span for the used experimental conditions: a vacuum environment ∼10^–5^ mbar and laser excitation power of 0.5 μW.^29−31^ The emission peak also survives multiple thermal cycles between room temperature and liquid helium temperature and remains around 1.704 eV. Jitter, characteristic for some localized excitons, is indeed present.^7,8,32,33^ It results from the fluctuation of the local electric field in the vicinity of the localized excitons and hence emission lines from the same emitter exhibit the same jitter pattern.^34,35^ The amplitude of the jitter is on the order of the line width or a few hundred microelectronvolts. The photoluminescence emission intensity of the localized excitons manifests a sublinear behavior with increasing laser power under high power excitation. Figure 2b displays the excitation power dependence of the integrated μ-PL intensity for the emission feature around 1.704 eV and, as anticipated, the dependence is sublinear when raising the laser power. This underlines the localized nature of the excitons responsible for the emission.^6,8,36^

In contrast to previous reports on uncontrolled impurity or defect-induced WSe_2_ quantum dots,^8,32^ no synchronized jitter of a doublet caused by electron–hole exchange interaction induced fine structure splitting is observed. We assert that the absence of a fine structure split doublet in the spectrum shown in Figure 2 is the result of the elongated strain potential along the edge of the substrate protrusion. This favors excitonic dipole oscillations along the edge, while penalizing or suppressing such oscillations in the perpendicular direction. The latter would produce an emission at higher energy due to the anisotropy induced fine structure splitting, but the emission is suppressed due to the lack of optical oscillator strength.^37−43^ For about 118 out of 208 localized exciton emission features studied, fine structure splitting was not observable because emission from the upper branch was suppressed so that it was not resolvable from the noise background. For the remaining 90 emission features, the emission from the upper branch was significantly suppressed as well but still observable. In comparison with the lower branch, the emission peak was 2.5–3% or less in strength (Section 5, SI). The predominant emission from excitonic dipole oscillations along the pillar edge is reminiscent of the exciton emission characteristics of nanowires with dipole emission exclusively along the wire axis.^44,45^ In view of this similarity, we refer to the emission observed here as quasi-1D exciton emission. For the sake of completeness, we note that photoluminescence spectra recorded on bubbles that form during rapid stacking of WSe_2_ monolayers from the same starting material on top of hexagonal boron nitride (hBN) do reveal doublet emission as shown in Section 6 of the SI. The weaker anisotropy of the strain-induced potential causes a smaller suppression of the upper branch emission. This was also the case for the strain-induced WSe_2_ quantum emitters of a previous report.^17^ We have also verified that the corners of the pillar geometry do not play an essential role by studying WSe_2_ monolayers placed on an array of wires with a width of 100 nm prepatterned on the silicon substrate covered with a dry thermal oxide (Section 7, SI). The results are essentially the same, but it is easier to locate localized excitons in the SiO_*x*_ pillar geometry. In the remainder, we therefore focus exclusively on data recorded on the square-shaped protrusions.

A characteristic feature of the emission in nanowires is linear polarization.^44,45^ Here, we anticipate that the emission of quasi-1D localized excitons should yield linear polarization as well, since the excitonic state is a linear superposition of K and K′ valley states of equal weight, , as discussed in more detail in Sections 8 and 9 of the SI.^46^Figure 2c,d shows the polarization of the emission peak around 1.704 eV. Panel c is a color rendition of the dependence of the spectrum on the polarization angle, whereas panel d displays the integrated photoluminescence intensity in a polar diagram with the angle representing the linear polarization direction. The emission is indeed linearly polarized. The degree of linear polarization can be quantified by using the expression , where *I*_max_ and *I*_min_ are the maximum and minimum intensity extracted from the polar diagram in Figure 2d. We find that *P*_linear_ = 95%. The linear polarization and its alignment along the edge of the substrate protrusion corroborates the quasi-1D nature of the localized excitons. The same mechanism also accounts for the linear polarization emission characteristics previously reported for the excitons from TMDs and their heterostructures created in strain potentials through various configurations: between gold nanorods, in elongated corrugations during the transfer of layers, due to random topography of the substrate, by a cantilever or a one-dimensional Moiré potential.^10,12,14,16,47^ The systematic control over the orientation of the linear polarization is demonstrated on an array of silicon-oxide pillars with different rotation angles in Section 10 of the SI.

**Figure 3:**
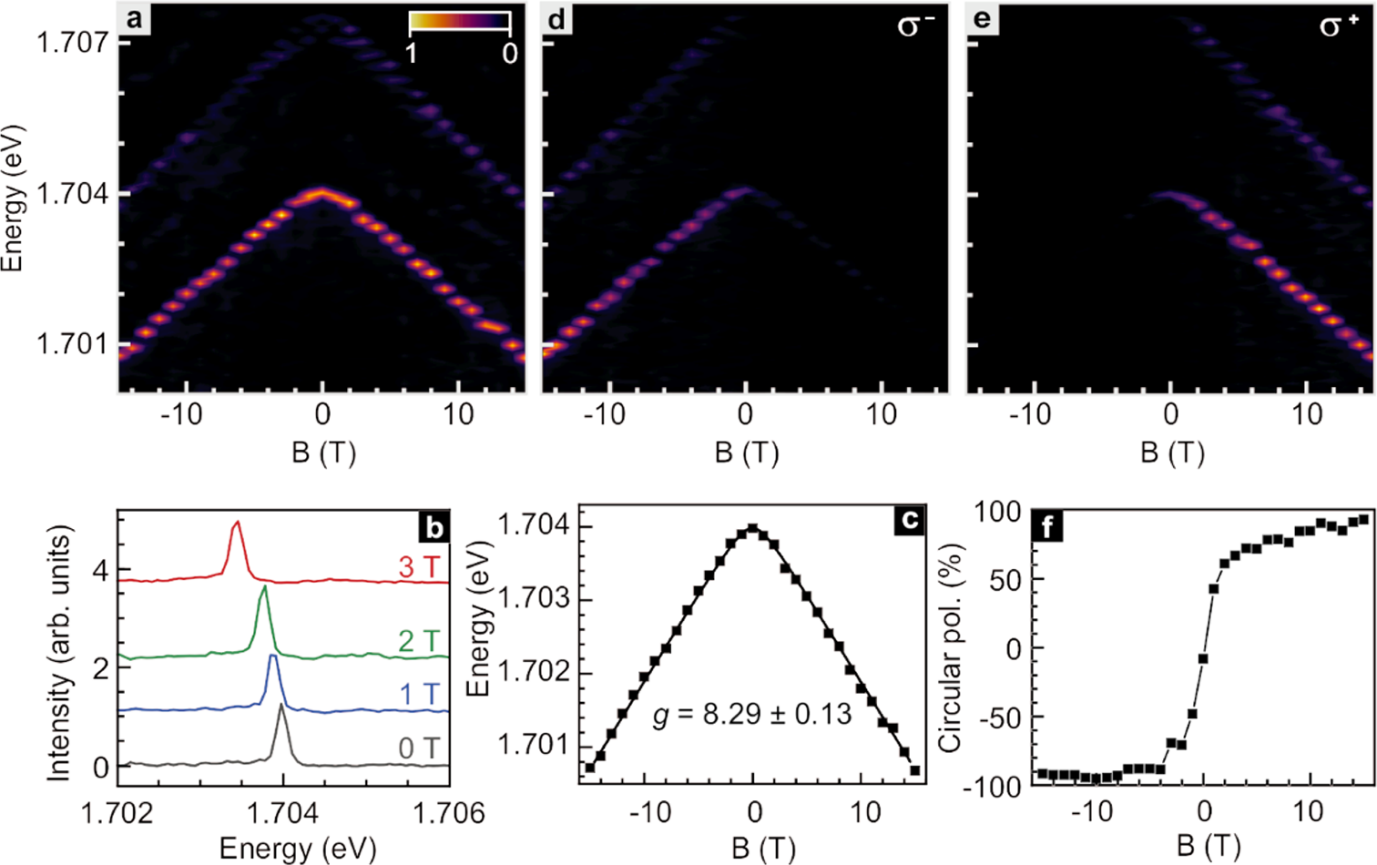
Magnetic field dependence of the μ-PL emission near 1.704 eV. (a) Polarization unresolved μ-PL emission as a function of magnetic field *B*. (b) Comparison of the μ-PL spectra around 1.704 eV recorded at 0 (black), 1 (blue), 2 (green), and 3 T (red). (c) Energy shift of the emission peak from the quasi-1D exciton as a function of *B*. The dots are experimental data points. The line is a fit to the data. (d) Left hand (σ^–^) and (e) right-hand (σ^+^) polarization-resolved μ-PL emission as a function of *B*. (f) Degree of circular polarization () as a function of *B*. In all color renditions, the normalized intensity is plotted.

The emission of WSe_2_ quantum dots in the presence of a magnetic field has been previously studied in the literature.^5−8,48,49^ It exhibits a strong Zeeman splitting, and the effective gyromagnetic ratio or Landé *g*-factor can be extracted from the data. Such a field-dependent study was also performed here, and the results are summarized in Figure 3. Panel a illustrates how the emission spectrum changes when varying the field between −15 and +15 T. With increasing magnetic field, the intensity of the emission remains nearly the same. Since the emission intensity of dark excitons strengthens with the magnetic field,^50,51^ it can be safely excluded that the localized excitons emission above 1.7 eV monitored here stems from dark excitons. A statistical study in ref (13) has identified that dark excitons have emission energies below 1.653 eV. It also corroborates the bright nature of the exciton studied here. In this manuscript, only emission features above 1.653 eV are considered that stem from localized bright excitons (Section 2, SI).^13^ The two emission peaks visible in Figure 3a shift with a nearly identical slope to lower energy when increasing the magnitude of the applied field |*B*|. No emission features are resolved that move up in energy with increasing field magnitude indicating that for each of the excitons the emission from the upper branch is suppressed in the entire magnetic field range covered by the measurements.

Figure 3b compares line traces recorded at a constant magnetic field of 0 (black), 1 (blue), 2 (green), and 3 T (red) for the exciton emitting near 1.704 eV. Figure 3c plots the dependence of the peak position for this emission feature with the applied *B*-field. The solid line is a fit to the data using the expression, . It enables the extraction of the Landé *g*-factor (*g*) and the zero-field fine structure splitting (δ_1_) caused by anisotropic electron–hole exchange interaction in the confinement potential that does not possess *D*_2*d*_ symmetry (see Sections 8 and 9, SI Figures S8 and S9). Here, μ_B_ is the Bohr magneton. The fit yields a Landé *g*-factor of 8.29 ± 0.13. This value is comparable with previously reported values for WSe_2_ quantum dots that varied between 7 and 13.^5−8,17^ For the zero-field fine structure splitting (δ_1_), we obtain 480.39 ± 7.24 μeV. This is less than previously reported values obtained for emission from excitons trapped at impurities or defects, which ranged from 670 to 770 μeV.^5,8^ In previous literatures,^43,52^ it has been discussed that the zero-field fine structure splitting depends on the asymmetry of the confinement potential but does not increase monotonously with increasing asymmetry. The splitting initially increases, but after crossing some threshold decreases again. The doublet emission from impurity/defect trapped excitons, discussed in refs (5) and (8), indicates only a small asymmetry of the confinement potential. In this work, the strong suppression or absence of emission from the upper energy branch confirms a strong asymmetry of the confining strain potential. Hence, we attribute the reduced zero-field fine structure splitting to the enhanced asymmetry in our geometry. The same experiments were repeated for the emission from excitons localized in WSe_2_ bubbles on the same starting material. This resulted in a Landé *g*-factor of 9.2 ± 0.05 and δ_1_ value of 577 ± 13 μeV (Section 11, SI). As discussed before, the fine structure split emission peak at higher energy is observed. This peak moves to higher energy with increasing magnetic field strength as anticipated, and the energy difference with respect to the lower energy branch reflects both the zero field fine structure splitting and the Zeeman splitting (Section 8, SI).

The application of a magnetic field gradually lifts the valley hybridization, that is, the initial linear superposition of K valley and K′ valley states of equal weight describing the quasi-1D localized excitons. As a result, the polarization of the emitted light should be converted from linear to circular polarization.^46^ The degree of circular polarization of the emission spectrum versus applied magnetic field was therefore investigated. These experimental results are presented in the panels of Figure 3. Figure 3d,e show color maps of the photoluminescence intensity in the plane spanned by the magnetic field and photon energy for left-hand (σ^–^) and right-hand (σ^+^) circularly polarized light emission near 1.704 eV, respectively. Examples of the recorded line traces at different magnetic fields can be found in Section 12 of the SI. For negative magnetic fields, the σ^–^ emission from the K′ valley is stronger. The same holds for the σ^+^ emission from the K valley for the positive magnetic field direction. This confirms in retrospect that the linear polarization of the localized exciton emission at zero magnetic field goes back to the excitonic state being an equal weight linear superposition of K and K′ valley states, . The degree of circular polarization, defined as , is plotted in Figure 3f as a function of magnetic field. It reaches more than 95% for field strengths exceeding 10 T, and of course the polarization has opposite chirality for opposite signs of the magnetic field.

**Figure 4:**
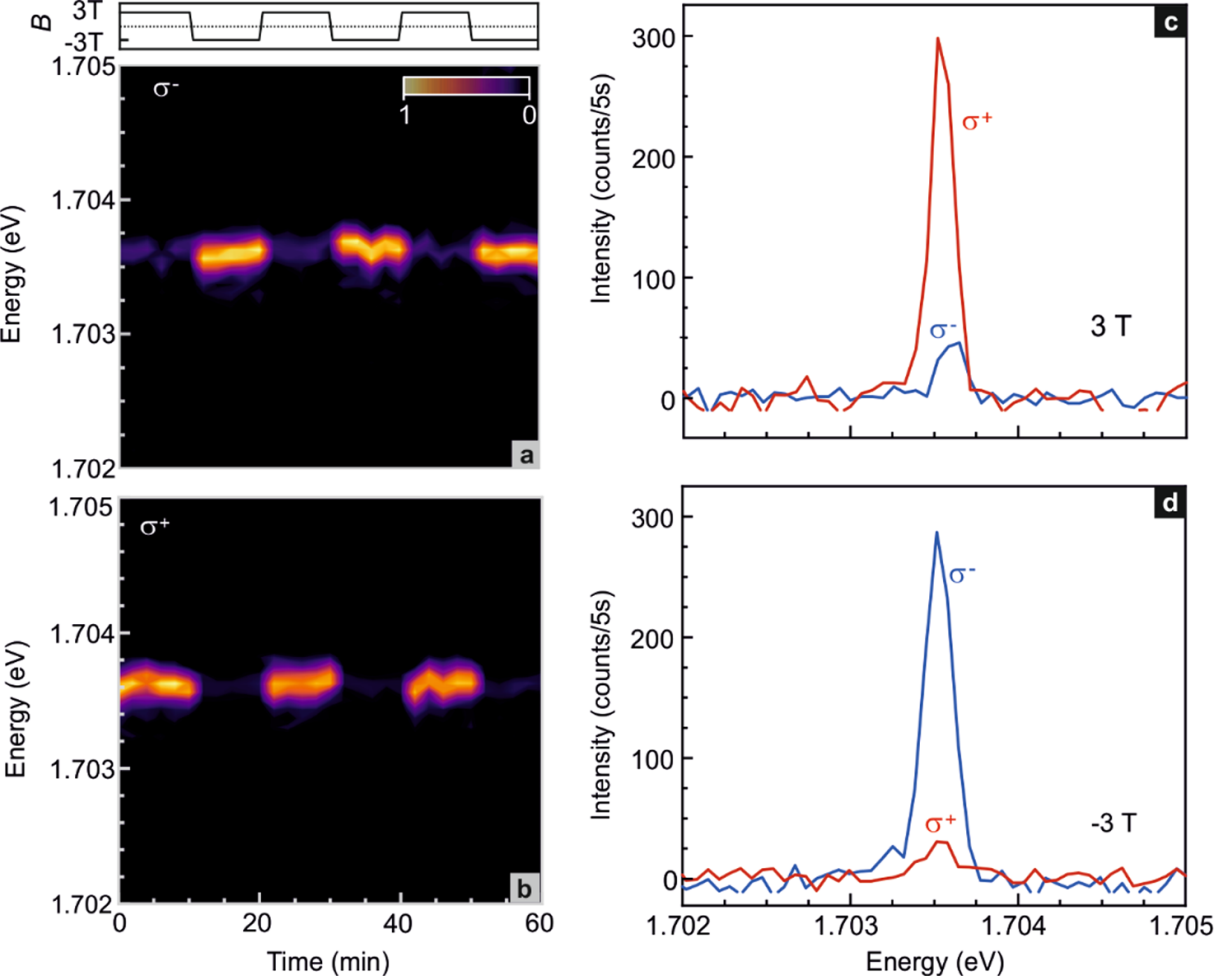
Tuning of the valley polarization by switching the magnetic field *B* between +3 and −3 T. (a) K′ valley (σ^–^) and (b) K valley (σ^+^) polarization-resolved μ-PL emission near 1.704 eV from the quasi-1D localized exciton upon periodic variation of the magnetic field between +3 and −3 T. (c,d) Comparison of the K′ valley (σ^–^) (blue) and K valley (σ^+^) (red) polarization resolved μ-PL spectra at 3 T (c) and −3 T (d).

Figure 4 depicts the emission behavior recorded while periodically changing the magnetic field between +3 T and −3 T in order to tune the valley polarization. No data are shown during the field sweeps. Panels a and b demonstrate that right-hand (σ^+^) circularly polarized emission from the K valley dominates at 3 T, while left-hand (σ^–^) circular polarized emission from the K′ valley is more intense at −3 T. Exemplary line traces for both fields and for both polarization directions are included in panels c and d.

## Conclusions

In conclusion, we have demonstrated that by cladding WSe_2_ on suitably designed substrate protrusions, it is possible to create a highly anisotropic, quasi-1D confinement potential for localizing excitons. Their emission spectrum is dominated by the low energy branch. The emission of the fine structure split upper energy branch can either not be resolved or is about 2 orders of magnitude smaller in strength. The emission in the absence of a field is linearly polarized with high purity (95%) due to an equal weight valley hybridization of the excitonic states and the suppressed emission from the higher energy exciton branch that would require dipole oscillations perpendicular to the edge of the square-shaped protrusion. Conversion to high purity circular polarization (95%) of either chirality is possible with the application of a strong magnetic field.

## Methods

### Sample Preparation

Our studies were performed on WSe_2_ monolayers subjected to spatially dependent strain either by placing the WSe_2_ layer intentionally on a substrate with a prepatterned array of protruding SiO_*x*_ pillars that act as local stressors (sample type I) or on top of exfoliated hBN flakes in a manner such that bubbles form (see below, sample type II). The SiO_*x*_ pillars with a square shape of 1 μm × 1 μm were fabricated on a Si substrate with a 300 nm thick dry thermal oxide. In a first step, an array of squares was patterned in PMMA resist with electron beam lithography. Subsequently, SiO_*x*_ with a thickness of 150 nm was deposited through thermal evaporation. The morphology of the remaining SiO_*x*_ pillars after lift-off of the SiO_*x*_ covered electron beam resist was characterized with the help of scanning electron microscopy (SEM) during the development phase of the sample fabrication procedure. Such test samples were sputter coated with a 4 nm thick layer of carbon to prevent charging during these SEM measurements. For measurement samples, the WSe_2_ monolayer, obtained via mechanically exfoliation from a bulk crystal (HQ-graphene), was transferred on part of the array of SiO_*x*_ pillars with a polydimethylsiloxane (PDMS) stamp based dry transfer method.^53−55^ In the type II samples, strain was generated by stacking the WSe_2_ monolayer, available on the PDMS stamp, with increased speed onto the hBN flake that was already transferred onto a thermally oxidized Si substrate. This rapid stacking promotes the formation of bubbles and hence local strain. Both sample types were annealed at 200 °C in a forming gas atmosphere H_2_/Ar (ratio 1:3) at 150 mbar for 5 h.

### Optical Measurements

Micro-photoluminescence (μ-PL) measurements were performed using a home-built confocal microscope inside a variable temperature insert of a cryostat equipped with an axial 15/17 T magnet. The sample was mounted in reflection geometry. For the magneto-luminescence experiments, the field is applied in the Faraday geometry perpendicular to the 2D layer. The sample was excited with a continuous-wave (CW) helium–neon laser at 632.8 nm. An excitation power of 0.5 μW was utilized unless otherwise specified. The photoluminescence signal was collected with a monochromator (iHR-320) equipped with a 1800 grooves/mm grating. A liquid nitrogen-cooled charge-coupled device (SynapsePlus BIDD CCD) served as detector. Linear polarization-resolved micro-photoluminescence measurements were performed by combining a half-wave (λ/2) plate and a linear polarizer in the collection path. Circular polarization-resolved micro-photoluminescence data were acquired by employing a quarter-wave (λ/4) plate and a linear polarizer or a calcite beam displacer in the collection path. Photon correlation measurements were carried out in a Hanbury–Brown and Twiss (HBT) setup at a temperature of ∼4 K collecting the emitted light with an objective with NA = 0.8. The excitation laser wavelength was 658 nm (CW) and two single photon counting modules from PerkinElmer were used.

## Supporting Information Available

SEM and optical microscope images; comparison of PL and Raman spectra on flat and strained WSe_2_; Lorentz function fit to the PL spectrum; second-order photon-correlation measurements; excitons with weak but resolvable upper energy branch emission; optical characterization of WSe_2_ monolayer bubbles in a WSe_2_/hBN van der Waals stack; localized exciton emission from WSe_2_ clad on protruding wires; model for excitons localized in a strain-induced oval shaped potential; models for quasi-1D excitons; silicon oxide pillars with different orientation; PL emission from localized excitons; jitter and *B*-dependence of the PL spectrum from excitons localized in a WSe_2_ bubble; circular polarization resolved PL spectra in the presence of a magnetic field (PDF)
