# Supplementary material for: Highly Polarized Single Photons from Strain-Induced Quasi-1D Localized Excitons in WSe2
Source: Nano Lett. 2021 Aug 23;21(17):7175–82. doi: 10.1021/acs.nanolett.1c01927 (PMC8431731; doi:10.1021/acs.nanolett.1c01927)
Supplement: Supplementary file 1 — nl1c01927_si_001.pdf [file nl1c01927_si_001.pdf]

Supplementary Information

Highly Polarized Single Photons from Strain Induced Quasi-1D Localized Excitons in WSe_2

*Qixing Wang[1], Julian Maisch[2], Fangdong Tang[1], Dong Zhao[1], Sheng Yang[1], Raphael Joos[2], Simone Luca Portalupi[2], Peter Michler[2] and Jurgen H. Smet[*1]*

[1]Max Planck Institute for Solid State Research, Stuttgart D-70569, Germany

[2]Institut für Halbleiteroptik und Funktionelle Grenzflächen, Center for Integrated Quantum Science and Technology (IQ^{ST}) and SCoPE, University of Stuttgart, Stuttgart, Germany

Corresponding author: Jurgen H. Smet (Email: j.smet@fkf.mpg.de, Tel: +49 711 689-5244)

Supplementary sections

S1. Scanning electron microscope and optical microscope images
S2. Comparison of photoluminescence and Raman spectra on flat and strained WSe_2
S3. Lorentz function fit to the PL spectrum
S4. Second-order photon-correlation measurements
S5. Excitons with weak but resolvable upper energy branch emission
S6. Optical characterization of WSe_2 monolayer bubbles in a WSe_2/hBN van der Waals stack
S7. Localized exciton emission from WSe_2 clad on protruding wires
S8. Model for excitons localized in a strain induced oval shaped potential
S9. Model for quasi-1D excitons
S10. Silicon-oxide pillars with different orientation
S11. Jitter and B-dependence of the PL spectrum from excitons localized in a WSe_2 bubble
S12. Circular polarization resolved PL spectra in the presence of a magnetic field

S1. Scanning electron microscope and optical microscope image

Figure S1a shows a SEM image of the substrate with a patterned array of protruding square shaped SiO_x pillars. Figure S1b displays an optical microscope image of a sample after a WSe_2 monolayer has been transferred on top of the array of pillars. Quasi-1D localized excitons form along the edges of the SiO_x pillars.

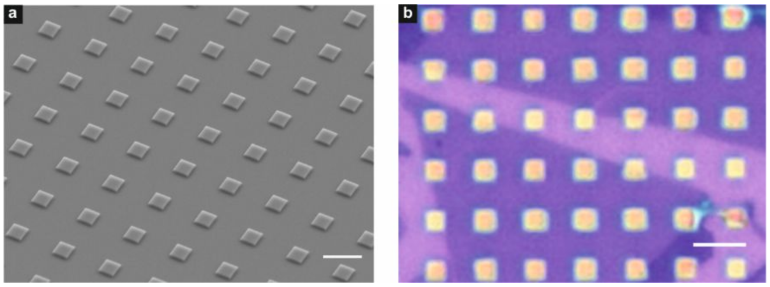

Figure S1. Scanning electron microscope and optical microscope image. (a) SEM image of an array of protruding square shaped SiO_x pillars. The dimension of one SiO_x pillar is approximately 1000 × 1000 × 150 nm. The white scale bar corresponds to 2 μm. (b) Optical microscope image of a sample where a WSe_2 monolayer is transferred on top of the array of pillars. The white scale bar is 3 μm long.

S2. Comparison of photoluminescence and Raman spectra on flat and strained WSe_2

Figure S2a shows the μ-PL spectrum of WSe_2 on a flat SiO_2 substrate. The peak at 1.75 eV is attributed to neutral exciton emission, while the peak at 1.68 eV corresponds to trion emission. Figure S2b compares the Raman spectrum of WSe_2 on flat SiO_2 (blue) with the spectrum of WSe_2 placed on a SiO_x pillar (red). The $E_{2g} + A_{1g}$ mode of WSe_2 on the SiO_x pillar is red shifted by about 0.2 cm^{-1}. This indicates that 0.5 % of strain is induced by the pillar.[1, 2] Figure S2c compares μ-PL spectra recorded on WSe_2 placed on a flat SiO_2 substrate (blue) and on a SiO_x pillar (red). Numerous sharp emission lines appear for the flake placed on top of the substrate protrusion. The sharp emission lines below 1.653 eV are attributed to localized dark excitons in view of the work reported in Ref[3]. Dark excitons in TMDCs possess a dipole moment that is oriented perpendicular to the plane of WSe_2 flakes, while for bright excitons the dipole moment is in the plane. Due to the orientation of the dipole, the detected emission intensity is normally weak.

However, due to the topography (pillars), part of the WSe_2 flake is oriented perpendicular to the substrate and the dipole moment of the dark excitons is parallel to the Si substrate plane. As a result, the collection efficiency of such localized dark excitons at the pillar edge can be as high as that for bright excitons. These dark excitons are not investigated any further in this manuscript. Figure S2d displays the μ-PL spectrum recorded on WSe_2 placed on a SiO_x pillar for different temperatures. Emission from shallow trapped excitons appears below 4 K.

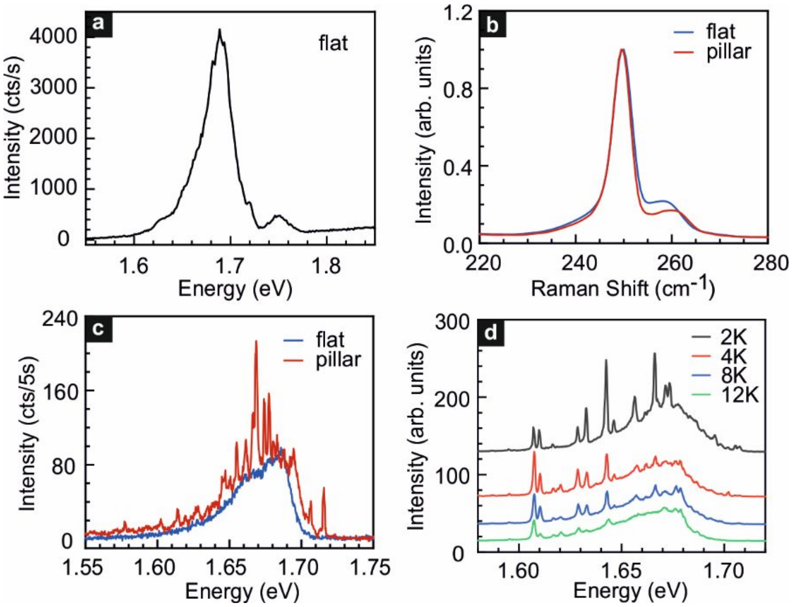

Figure S2. Comparison of photoluminesence and Raman spectra recorded on flat and strained WSe_2. (a) μ-PL spectra of WSe_2 placed on a flat SiO_2 substrate for 400 μW laser excitation at 2 K. The monochromator grating has 150 grooves/mm. (b) A comparison of the normalized Raman spectra recorded on WSe_2 on flat SiO_2 (blue) and on a SiO_x pillar (red). Raman measurements were performed at room temperature for 532 nm CW laser excitation. (c) Comparison of the μ-PL spectra of WSe_2 on flat SiO_2 (blue) and on a SiO_x pillar (red) at 2 K. The monochromator grating has 600 grooves/mm. The excitation power is 0.1 μW. (d) μ-PL spectra recorded on WSe_2 placed on a SiO_x pillar for different temperatures: 2 K (black), 4 K (red), 8 K (blue), and 12 K (green). The spectra were vertically offset for the sake of comparison. The monochromator grating has 600 grooves/mm. The excitation power is 0.1 μW.

S3. Lorentz function fit to the PL spectrum

Figure S3 displays a Lorentz function fit of the peak marked with the blue dot in Figure 1b of the main text. The maximum is located at 1.704 eV and the peak has a full width at half maximum (FWHM) of 160 ± 14 μeV.

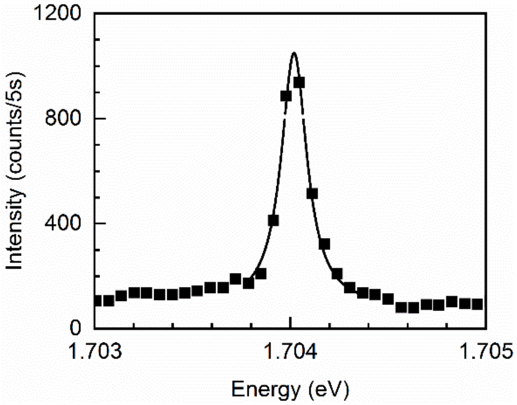

Figure S3. Lorentz function fit to the μ-PL peak marked in Figure 1b of the main text.

S4. Second-order photon-correlation measurements

Second-order photon-correlation measurements were performed by using a Hanbury Brown and Twiss (HBT) setup. The experimental data were fitted with the second-order photon-correlation function

$$g^{(2)}(t) = 1 - (1-a)e^{-|t|/\tau} \quad (1)$$

where t is the time delay between the coincidence counts, τ consists of pumping and radiative recombination rate, and a represents the value of $g^{(2)}(t=0)$. In some cases, bunching behaviour is observed during second-order photon-correlation measurements. To include the bunching behavior visible in such cases, Eq. (1) is extended to

$$g^{(2)}(t) = (1 - (1-\tilde{a})e^{-\frac{|t|}{\tilde{\tau}}})(1 + be^{-\frac{|t|}{\tau_{bunch}}}) \quad (2)$$

where $\tilde{\tau}$ and τ_{bunch} consist of pumping and radiative recombination rates of the anti-bunching and bunching components, respectively, $\tilde{a}$ and b are constants. Figure S4 presents the $g^{(2)}(t)$ results from another emitter where bunching is superimposed to the anti-bunching. The data points are fitted with Eq. (2). At $t = 0$, $g^{(2)}(0) = \tilde{a}(1+b) = 0.27 \pm 0.09 < 0.5$. The extracted values from the fit are $\tilde{\tau} = 12.03 \pm 2.58$ ns and $\tau_{bunch} = 9.06 \pm 0.85$ ns.

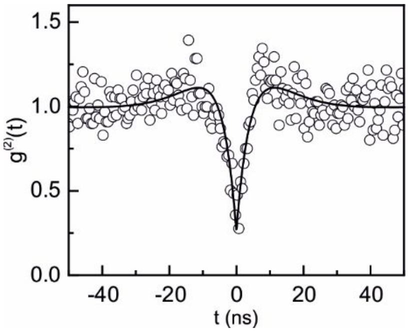

Figure S4. Second-order photon-correlation results for an emitter with bunching. The data points are fitted with Eq. (2).

S5. Excitons with weak but resolvable upper energy branch emission

The PL spectrum in Figure S5a shows an example for emission from a localized exciton where the upper energy branch also contributes a small luminescence peak. The peak intensity ratio of the lower branch to upper branch is about 40 : 1. The lower branch and upper branch emission are cross-linearly polarization (Figure S5b). The polarization direction of the lower branch remains parallel to the edge of the pillar.

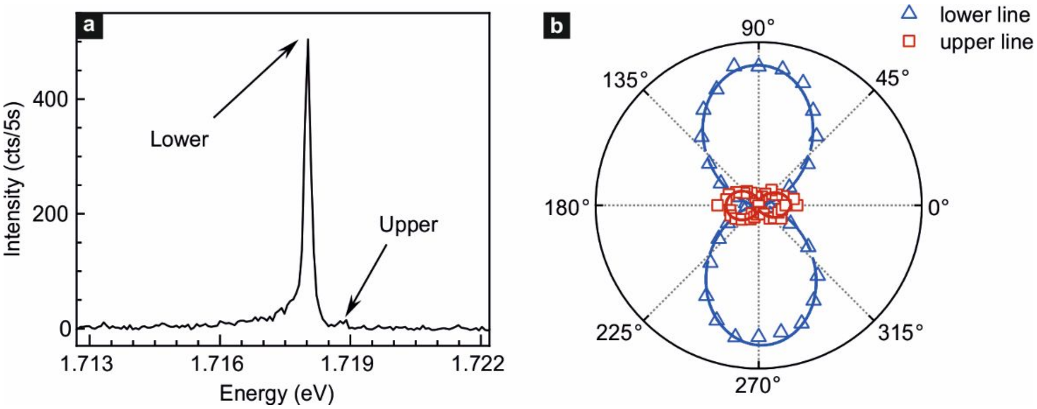

Figure S5. Emission from a localized exciton with weak but resolvable upper energy branch emission. (a) PL spectrum. (b) Integrated μ-PL intensity for both emission features in panel a plotted as a function of the detection angle in a polar diagram. The solid lines are fits to the data sets with sine functions. The intensity of the upper branch emission line is multiplied by five for ease of comparison.

S6. Optical characterization of WSe_2 monolayer bubbles in a WSe_2/hBN van der Waals stack

Figure S6a shows an optical microscope image of WSe_2 monolayer bubbles that form when rapidly assembling a WSe_2/hBN stack. The PL spectrum from localized excitons in the WSe_2 bubble 1 is plotted in Figure S6b. Fine structure splitting (FSS) is observed. This splitting is attributed to an anisotropic dot confinement potential which is accompanied by anisotropic electron-hole exchange interaction.[4-6] The synchronized jitter in panel c of the lower and upper photoluminescence peaks indicates that both originate from the same quantum dot confinement potential (Figure S6c). The emission for both is linearly polarized. Moreover, the emitted light is cross-polarized as illustrated in Figure S6d and e.

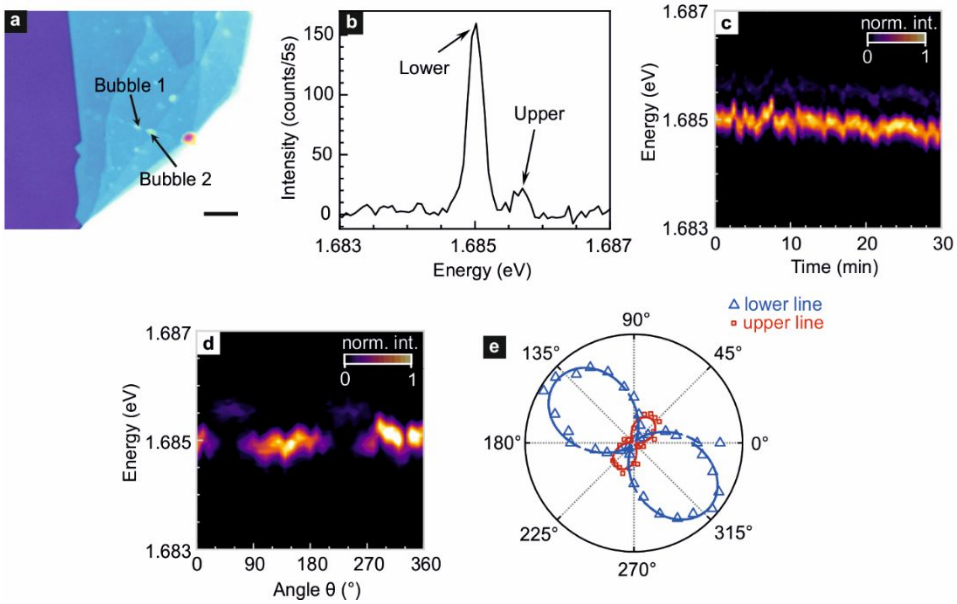

Figure S6. Optical characterization of WSe_2 monolayer bubbles on hBN. (a) Optical microscope image of a WSe_2 monolayer placed rapidly on top of a hBN flake so that bubbles form. The black scale bar corresponds to 5 μm. (b) μ-PL spectrum of the localized exciton in the WSe_2 bubble marked as "Bubble 1" in panel (a). Fine structure splitting produces two peaks in the spectrum. The ratio of the peak intensity is about 7 : 1. (c) μ-PL emission from the same bubble recorded as a function of time to analyze the jitter. (d) Linear polarization resolved μ-PL as a function of detection angle. The color bar refers to the normalized μ-PL intensity. (e) Integrated μ-PL intensity for both emission features (blue triangles for the lower energy and red squares for the emission at higher energy) as a function of detection angle. The intensity of the upper emission line is

multiplied by two for ease of comparison. Solid lines are best fits to the data using the expression $I = I_0 + b\sin\frac{\pi(\theta - \theta_0)}{a}$.

S7. Localized exciton emission from WSe_2 clad on protruding wires

To verify whether the corners of the square shaped protrusions have any relevant impact on the emission characteristics of the localized excitons, also exciton emission on the geometry illustrated in Figure S7a was investigated. This panel shows a scanning electron microscope (SEM) image of an array of SiO_x wires each with a width of 100 nm in order to avoid any influence from corners. WSe_2 monolayers are transferred on top of the wires to create a one-dimensional strain potential for the confinement of excitons (Figure S7b). A typical μ-PL spectrum emitted at one site of the wires is depicted in Figure S7c. The emission is linearly polarized and the polarization orientation is parallel to the wires. We conclude that the emission characteristics are similar and the corners in the square shaped protrusions of the samples studied in the main text have no significant influence for the studied exciton emission.

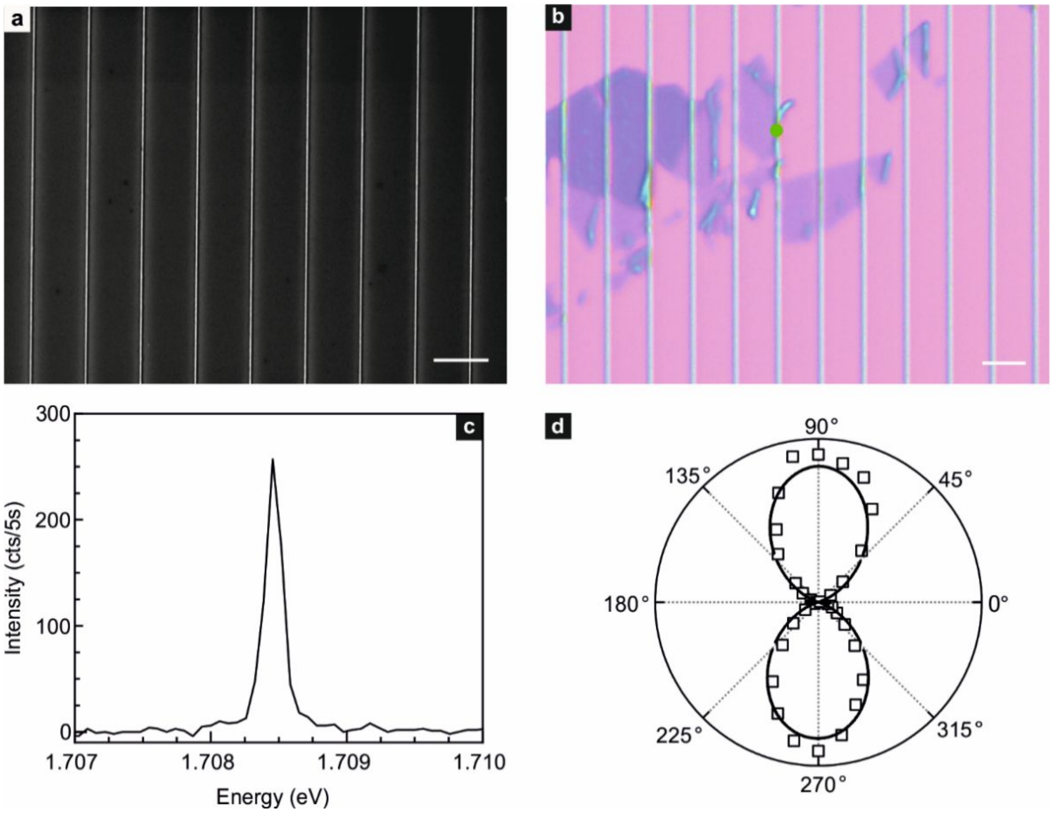

Figure S7. Wire sample image and emission characteristics of a localized exciton. (a) SEM image of an array of protruding SiO_x wires, each with a width of 100 nm and a height of 150 nm. The white scale bar corresponds to 3 μm. (b) Optical microscope image of a sample where a WSe_2 monolayer is placed on top of an array of wires. The white scale bar is also 3 μm long. (c) μ-PL spectrum from a localized exciton. The location is marked with a green dot in panel b. (d) Integrated μ-PL intensity of the exciton peak in panel c plotted as a function of the detection angle in a polar diagram. The solid line is a fit to the data with a sine function.

S8. Model for excitons localized in a strain induced oval shaped potential

The model of quasi-1D localized excitons in an elongated strain induced potential has been deduced from the established model of excitons subjected to an anisotropic confinement potential.[4-6] Therefore, the latter case will be treated here first.

For a WSe_2 monolayer, the valley and spin degrees of freedom are locked. Hence, optical emission from the K and K' valleys are right (σ^+) and left hand (σ^-) circularly polarized, respectively. It is therefore convenient to use the notation $|K\rangle$ and $|K'\rangle$ to represent excitonic states that emit right and left hand circularly polarized light. For the sake of simplicity, we will assume an oval shaped confinement potential without D_{2d} symmetry, i.e. the *x* and *y* in-plane directions are not equivalent.[4-6] In such a potential, the excitonic states possess a dipole moment that is either oriented along the minor or major axis of the oval shape. The minor axis is referred to as the *x*-direction, whereas the major axis is aligned along the *y*-direction as shown schematically in panel a of Figure S8. The energy of the exciton with its dipole moment aligned along the *x*-direction is higher because of the tighter quantum confinement. The photoluminescence intensity from this higher energy exciton in *x*-direction is lower because of the smaller optical oscillator strength in the direction of the minor axis.[7-13] The eigenstates at zero magnetic field (B = 0) are a linear superposition of the localized $|K\rangle$ and $|K'\rangle$ exciton states with equal weight and therefore can be written as

$$|X_L\rangle = \frac{1}{\sqrt{2}}\left(|K\rangle - |K'\rangle\right),$$

$$|X_U\rangle = \frac{1}{\sqrt{2}}\left(|K\rangle + |K'\rangle\right).$$

The index L refers to the exciton with lower energy, whereas U is used for the upper, higher lying exciton. These energies are given by

$$E_L = E_0 + \frac{1}{2}\delta_0 - \frac{1}{2}\delta_{1,}$$

$$E_U = E_0 + \frac{1}{2}\delta_0 + \frac{1}{2}\delta_{1.}$$

Here, δ_0 is the zero-field splitting between radiative and dark excitons and δ_1 is the zero-field fine structure splitting caused by anisotropic electron–hole exchange interaction in the confinement potential without D_{2d} symmetry (Figure S8b).

At non-zero magnetic field ($B \neq 0$), the eigenstates are no longer an equal weight linear superposition of the two valley states. One valley is favored over the other depending on the sign of the magnetic field. The following equations describe the lower and upper excitonic state and their energies when applying an external magnetic field:

$$|X_L\rangle = N_L\left(|K\rangle + \left(\alpha - \sqrt{1+\alpha^2}\right)|K'\rangle\right),$$

$$|X_U\rangle = N_U\left(|K\rangle + \left(\alpha + \sqrt{1+\alpha^2}\right)|K'\rangle\right),$$

$$E_L = E_0 + \frac{1}{2}\delta_0 - \frac{1}{2}\sqrt{{\delta_1}^2 + (g\mu_B B)^2},$$

$$E_U = E_0 + \frac{1}{2}\delta_0 + \frac{1}{2}\sqrt{{\delta_1}^2 + (g\mu_B B)^2}.$$

In these expressions, α is equal to $\frac{g\mu_B B}{\delta_1}$, μ_B is the Bohr magneton, g the Landé or gyromagnetic factor, and N_L and N_U are magnetic field dependent normalization constants. The energy difference between the high energy state and the low energy state is obtained from

$$\Delta(E) = E_U - E_L = \sqrt{{\delta_1}^2 + (g\mu_B B)^2}.$$

For strong magnetic fields, when $\alpha = \frac{g\mu_B B}{\delta_1} \gg 1$, $|X_L\rangle = |K\rangle$ and $|X_U\rangle = |K'\rangle$, and vice versa for the opposite sign of the magnetic field ($\alpha \ll -1$, $|X_L\rangle = |K'\rangle$ and $|X_U\rangle = |K\rangle$). The expressions for the energies reduce to

$$E_L = E_0 + \frac{1}{2}\delta_0 - \frac{1}{2}g\mu_B|B|,$$

$$E_U = E_0 + \frac{1}{2}\delta_0 + \frac{1}{2}g\mu_B|B|.$$

The key takeaways from this model are summarized in Figure S8b. The anisotropy in the strain-induced confinement potential results in a fine structure splitting δ_I in the absence of a magnetic field between the two possible exciton states whose emissions exhibit linear and cross polarization. In the presence of a magnetic field, the fine structure splitting acquires a Zeeman term and varies according to $\Delta(E) = \sqrt{{\delta_1}^2 + (g\mu_B B)^2}$.

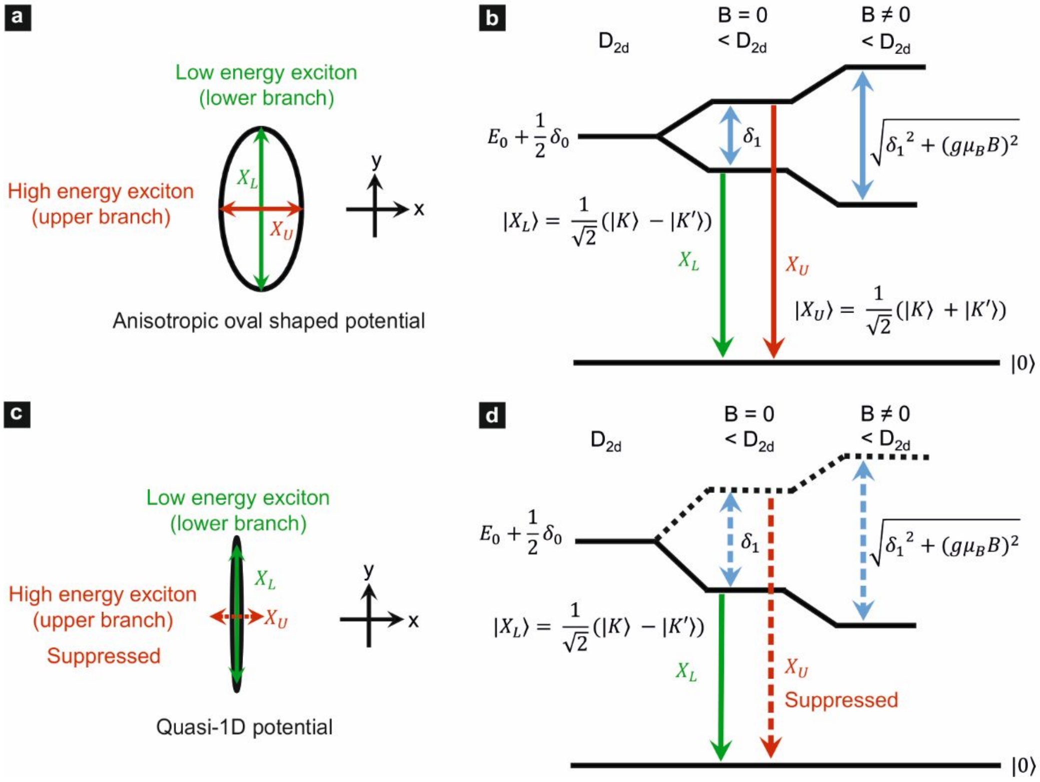

Figure S8. Excitons and their energy in the absence and presence of a magnetic field for a quantum dot with a strain induced oval shaped or a quasi-1D confinement potential. (a) Schematic illustration of the oval shaped confinement potential as it may occur in real space, for example, in bubbles that form in WSe_2 monolayer when stacked rapidly on top of hBN. (b) Schematic of the energy level diagram illustrating the fine structure splitting of localized excitons in the absence and presence of a magnetic field. δ_0 refers to the zero-field splitting between radiative and dark excitons. δ_1 is the zero-field fine structure splitting due to an anisotropy in the electron–hole exchange interaction in the oval shaped confinement potential. (c) A schematic illustration of the strain induced quasi-1D confinement potential with large anisotropy between the major and minor axis for a WSe_2 monolayer along the edge of a square shaped SiO_x pillar. Only dipole oscillations along the elongated confinement direction, i.e. the *y*-direction, can be expected. (d) Corresponding energy level diagram for quasi-1D excitons in the absence and presence of a magnetic field. The strongly suppressed optical transitions from the higher energy exciton state to the ground state has been marked with dashed lines.

S9. Model for quasi-1D excitons

We refer to quasi-1D excitons for the case where the ratio of the length of the major axis to the length of the minor axis of the anisotropic strain induced confinement potential approaches infinity. This case of extreme anisotropy is illustrated in Figure S8c. While the expressions for the excitonic states and energies discussed in Section 8 still hold, optical transitions along the *x*-direction with its very tight confinement are strongly suppressed and only the low energy exciton state $|X_L\rangle$ with the dipole oriented along the *y*-direction can be considered optically active because of the lack of optical oscillator strength in the direction of the minor axis.[7-13] The emission of the upper branch is therefore strongly suppressed. The emission properties of $|X_L\rangle$ remain unaltered, i.e. linearly polarized in the absence of a magnetic field, while the polarization is converted from linear to circular for strong magnetic fields.

S10. Silicon-oxide pillars with different orientation

The linear polarization of the emission of localized excitons from WSe_2 clad onto the square shaped protrusion is aligned parallel to the edge where the emission appears. Hence the orientation of the linear polarization is fully controlled by geometry. This is illustrated clearly in Figure S9 on a sample where the orientation of the square shaped pillars is systematically altered. Pillars in each of the rows have the same orientation, however the pillars of adjacent rows are rotated by 10° with respect to each other. The angle of the pillars starts at 0° for the bottom row and ends at 90° for the top row. Figure S9a shows the SEM image of such a sample. Figure S9b displays a microscope image after placing a WSe_2 monolayer on top of this pre-patterned substrate with pillars. Typical PL emission spectra recorded at the edge of pillars with an angle of 10°, 50° and 90° are plotted in Figure S9c. The polarization direction of the emission is verified by plotting the integrated μ-photoluminescence intensity as a function of the detection angle in a polar diagram (Figure S9d). As anticipated, the orientation follows the rotation angle of the pillar. The locations where the different data sets were recorded are marked by colored dots in Figure S9b.

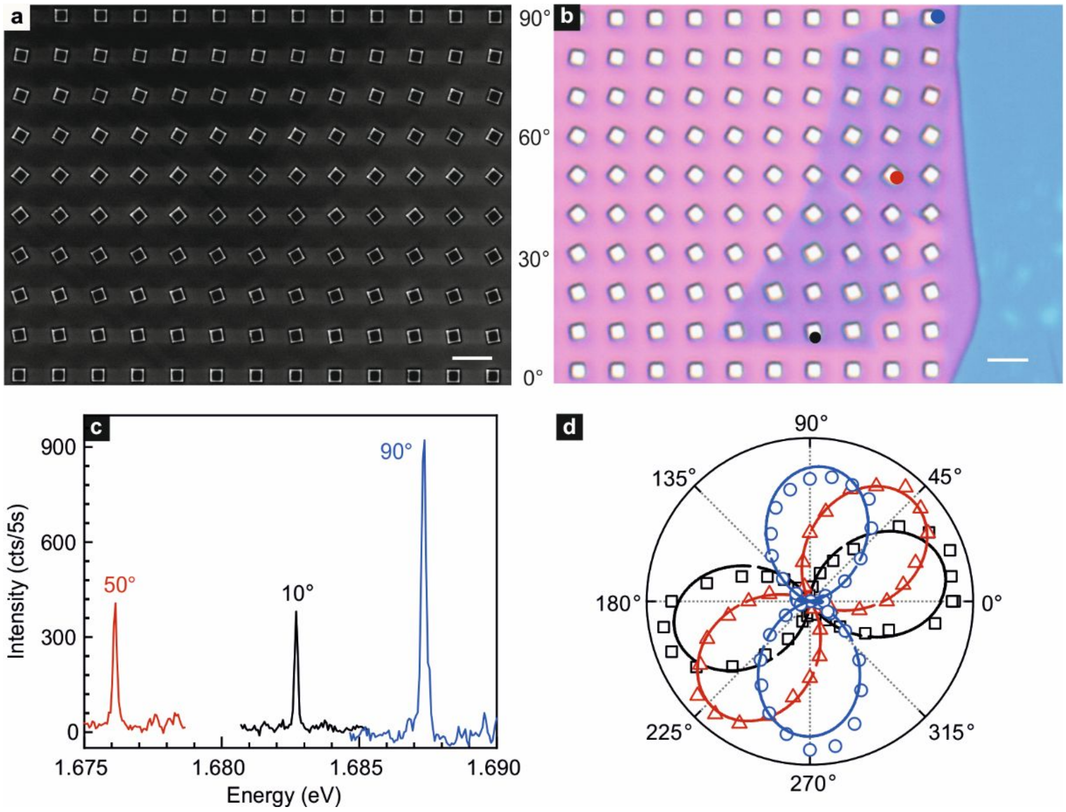

Figure S9. Silicon-oxide pillars with varying rotation angles. (a) SEM image of the array of protruding square shaped SiO_x pillars. Pillars of adjacent rows are rotated by 10° with respect to each other: top row 90° bottom row 0°. The white scale bar corresponds to 3 μm. (b) Optical microscope image of the sample after a WSe_2 monolayer has been transferred on top of the array. The white scale bar again equals 3 μm. (c) μ-PL spectra showing emission peaks from localized excitons for pillars with different rotation angles: 10° (black), 50° (red), and 90° (blue). Locations on the sample where these data were recorded are marked with black, red, and blue dots in panel b. (d) Integrated μ-PL intensity of the excitons in panel c plotted as a function of the detection angle in a polar diagram. The solid lines are fits to the data sets with sine functions.

S11. Jitter and B-dependence of the PL spectrum from excitons localized in a WSe_2 bubble

Figure S10a illustrates the photoluminescence (PL) spectrum recorded from Bubble 2 (See Figure S6a) that has formed in the WSe_2 monolayer after it was placed on top of hBN. The photoluminescence exhibits two peaks whose energy positions jitter in a synchronized manner when recording the spectrum over an extended period of time (Figure S10b). We therefore conclude that both emission features stem from excitons localized within the same confinement potential. With the increase of $|B|$, the two luminescence peaks split further in energy. The one with higher energy moves upward, while the one with lower energy moves downward (Figure S10c, d, and e). The energy difference between the upper branch and lower branch increases with the increase of $|B|$ and can be fitted by the equation $\Delta(E) = \sqrt{{\delta_1}^2 + (g\mu_B B)^2}$. The fitted zero field fine structure splitting, δ_1, is approximately 577 ± 13 μeV and the extracted g-factor equals 9.17 ± 0.04 (Figure S10f). With the increase of B, the PL intensity of the upper branch quenches gradually because excitons have a higher probability to fill the lower energy branch as the energy splitting increases.

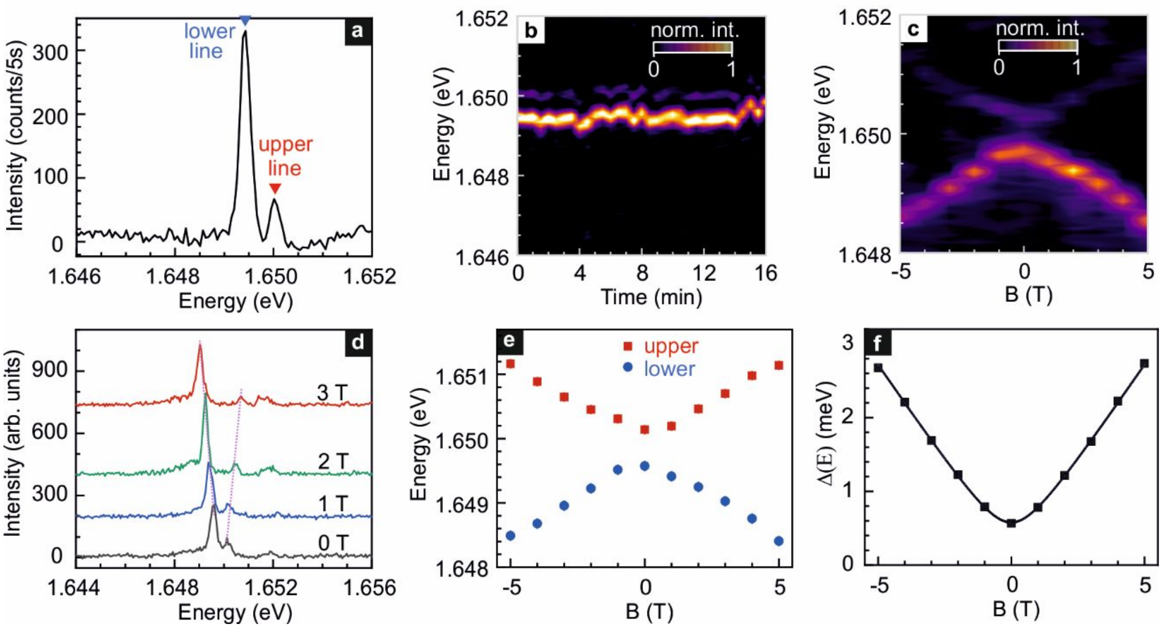

Figure S10. PL emission from a WSe_2 monolayer bubble. (a) μ-PL spectrum acquired on WSe_2 Bubble 2 in Figure S6a. (b) Time dependent record of the μ-PL. The color bar refers to the normalized μ-PL intensity. (c) Magnetic field dependence of the μ-PL spectrum. The polarization is not resolved in this experiment. (d) Line traces recorded at a magnetic field of 0 T (black), 1 T (blue), 2 T (green), and 3 T (red). (e) The magnetic field dependence of the peak positions in the luminescence spectrum. (f) Energy difference between the upper and lower branch emission peaks as a function of magnetic field B. The line is a fit to the data points using the function $\Delta(E) = \sqrt{{\delta_1}^2 + (g\mu_B B)^2}$.

S12. Circular polarization resolved PL spectra in the presence of a magnetic field

Figure S11 illustrates the magnetic field dependence of the circular polarization resolved PL spectra (left hand circular polarization σ^- and right hand circular polarization σ^+) from the localized excitons emitting at 1.704 eV as discussed in the main text. The data sets shown are for 0 T, ±2 T, ±5 T, and ±10 T.

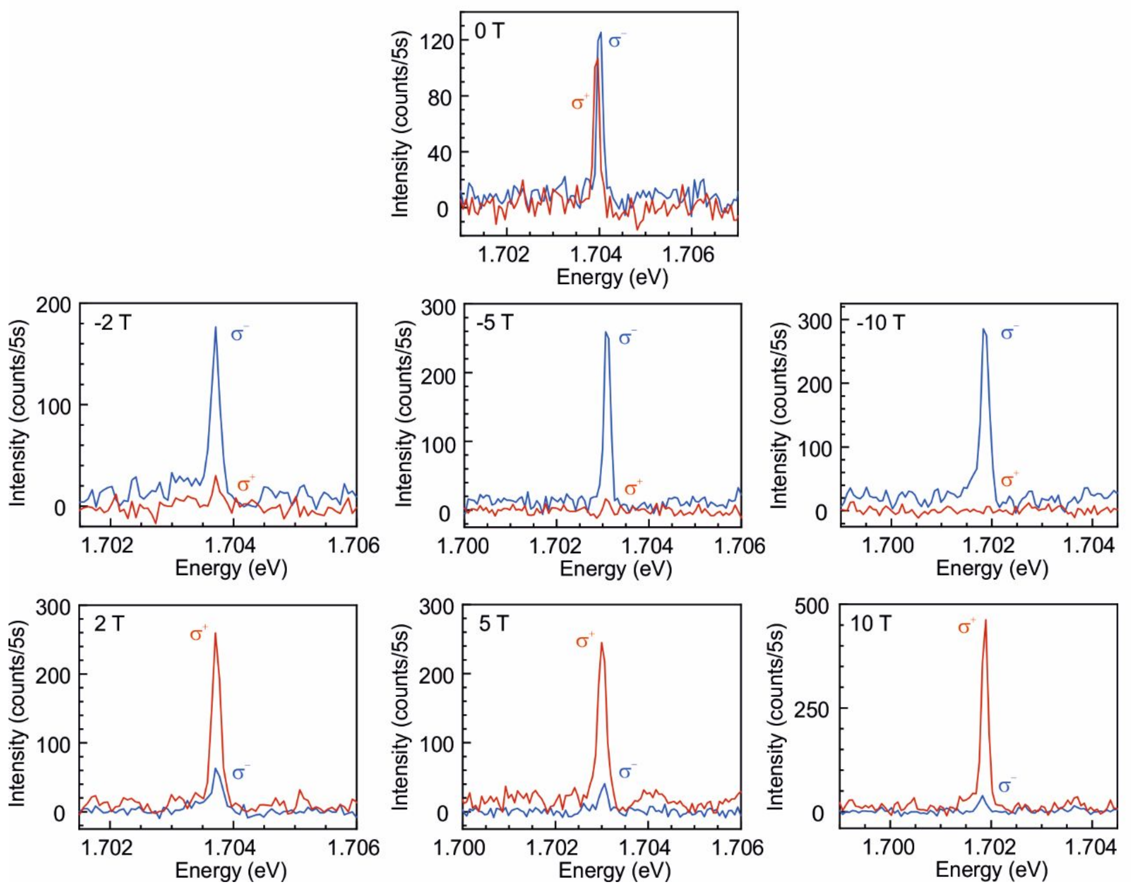

Figure S11. Circular polarization resolved μ-PL spectra for quasi-1D localized excitons emitting at 1.704 eV recorded at different perpendicular magnetic field strengths. Blue lines correspond to left-hand circular polarization (σ^-), whereas data for right-hand polarization (σ^+) are plotted in red. The magnetic field is equal to 0 T, ±2 T, ±5 T, or ±10 T.

REFERENCES

(1) Desai, S. B.; Seol, G.; Kang, J. S.; Fang, H.; Battaglia, C.; Kapadia, R.; Ager, J. W.; Guo, J.; Javey, A. Strain-Induced Indirect to Direct Bandgap Transition in Multilayer WSe_2. *Nano Lett.* **2014,** *14*, 4592-4597.
(2) Dadgar, A. M.; Scullion, D.; Kang, K.; Esposito, D.; Yang, E. H.; Herman, I. P.; Pimenta, M. A.; Santos, E. J. G.; Pasupathy, A. N. Strain Engineering and Raman Spectroscopy of Monolayer Transition Metal Dichalcogenides. *Chemistry of Materials* **2018,** *30*, 5148-5155.
(3) Luo, Y.; Liu, N.; Kim, B.; Hone, J.; Strauf, S. Exciton Dipole Orientation of Strain-Induced Quantum Emitters in WSe_2. *Nano Lett.* **2020,** *20*, 5119-5126.
(4) Finley, J. J.; Mowbray, D. J.; Skolnick, M. S.; Ashmore, A. D.; Baker, C.; Monte, A. F. G.; Hopkinson, M. Fine Structure of Charged and Neutral Excitons in InAs-$Al_{0.6}Ga_{0.4}As$ Quantum Dots. *Phys. Rev. B* **2002,** *66*, 153316.
(5) Kulakovskii, V. D.; Bacher, G.; Weigand, R.; Kümmell, T.; Forchel, A.; Borovitskaya, E.; Leonardi, K.; Hommel, D. Fine Structure of Biexciton Emission in Symmetric and Asymmetric CdSe/ZnSe Single Quantum Dots. *Phys. Rev. Lett.* **1999,** *82*, 1780-1783.

(6) Bayer, M.; Ortner, G.; Stern, O.; Kuther, A.; Gorbunov, A. A.; Forchel, A.; Hawrylak, P.; Fafard, S.; Hinzer, K.; Reinecke, T. L.; Walck, S. N.; Reithmaier, J. P.; Klopf, F.; Schäfer, F. Fine Structure of Neutral and Charged Excitons in Self-Assembled In(Ga)As/(Al)GaAs Quantum Dots. *Phys. Rev. B* **2002,** *65*, 195315.
(7) Vouilloz, F.; Oberli, D. Y.; Dupertuis, M.-A.; Gustafsson, A.; Reinhardt, F.; Kapon, E. Effect of Lateral Confinement on Valence-Band Mixing and Polarization Anisotropy in Quantum Wires. *Phys. Rev. B* **1998,** *57*, 12378.
(8) Besombes, L.; Kheng, K.; Martrou, D. Exciton and Biexciton Fine Structure in Single Elongated Islands Grown on a Vicinal Surface. *Phys. Rev. Lett.* **2000,** *85*, 425.
(9) Bockelmann, U.; Bastard, G. Interband Absorption in Quantum Wires. I. Zero-Magnetic-Field Case. *Phys. Rev. B* **1992,** *45*, 1688.
(10) Tanaka, T.; Singh, J.; Arakawa, Y.; Bhattacharya, P. Near Band Edge Polarization Dependence as a Probe of Structural Symmetry in GaAs/AlGaAs Quantum Dot Structures. *Appl. Phys. Lett.* **1993,** *62*, 756-758.
(11) Bardoux, R.; Guillet, T.; Gil, B.; Lefebvre, P.; Bretagnon, T.; Taliercio, T.; Rousset, S.; Semond, F. Polarized Emission from GaN/AlN Quantum Dots: Single-Dot Spectroscopy and Symmetry-Based Theory. *Phys. Rev. B* **2008,** *77*, 235315.
(12) Winkelnkemper, M.; Seguin, R.; Rodt, S.; Schliwa, A.; Reissmann, L.; Strittmatter, A.; Hoffmann, A.; Bimberg, D. Polarized Emission Lines from A- and B-Type Excitonic Complexes in Single InGaN/GaN Quantum Dots. *J. Appl. Phys.* **2007,** *101*, 113708.
(13) Zieliński, M. Vanishing Fine Structure Splitting in Highly Asymmetric InAs/InP Quantum Dots without Wetting Layer. *Sci. Rep.* **2020,** *10*, 13542.
